# Special immune-related adverse events and subsequent photodynamic therapy in tislelizumab treatment for esophageal cancer: a case report

**DOI:** 10.3389/fimmu.2024.1497259

**Published:** 2024-11-25

**Authors:** Longzhao Li, Lingjie Bian, Na Kou, Yue Yuan, Heng Zou

**Affiliations:** ^1^ Respiratory Disease Center, Dongzhimen Hospital, Beijing University of Chinese Medicine, Beijing, China; ^2^ Integrative Traditional Chinese and Western Medicine, Graduate School, Beijing University of Chinese Medicine, Beijing, China

**Keywords:** esophageal cancer, Tislelizumab, immune-related adverse events, photodynamic therapy, PD-1 inhibitors

## Abstract

This case report highlights the immune-related adverse events (irAEs) that occurred during the treatment of esophageal cancer with Tislelizumab and discusses management strategies, indicating that photodynamic therapy (PDT) may be an optimal adjunctive treatment option. Following Tislelizumab therapy, the patient demonstrated significant tumor reduction; however, subsequent irAEs related to immunotherapy emerged, including eyelid muscle weakness and myocardial and skeletal muscle injury. Methylprednisolone successfully alleviated these symptoms, with early intervention being crucial for controlling irAEs. The patient then underwent PDT, which not only further helped manage irAEs but also inhibited tumor progression. This case underscores the specific adverse reactions, such as eyelid ptosis, skeletal muscle, and myocardial damage associated with Tislelizumab, and the importance of early corticosteroid intervention. It also emphasizes the significance of PDT as an adjunctive treatment for controlling tumors and alleviating immune-related adverse reactions.

## Introduction

Immune checkpoint inhibitors have widespread clinical applications and have demonstrated durable antitumor responses across various cancer types (1). However, these agents can inadvertently activate non-tumor-specific T cells, leading to a range of irAEs affecting multiple organs. Commonly affected systems include the lungs, heart, skin, and gastrointestinal tract (2). Due to the non-specific nature of irAEs, diagnosis, and differentiation in clinical practice can be challenging.

Tislelizumab is a classic immune PD-1 checkpoint inhibitor that plays a significant role in the treatment of various cancers, including lung cancer, esophageal cancer, and hepatocellular carcinoma. For Tislelizumab, common immune-related adverse reactions include pneumonia, hypothyroidism, arthralgia, and vitiligo (3). Cardiac, ocular muscle neurological, and skeletal muscle toxicities are relatively rare, and there have been no reported cases of ocular muscle damage.

When severe adverse reactions occur with immune checkpoint inhibitors, the next steps in treatment are often controversial. Given the significant synergistic effects of PDT with anti-PD-1 agents, which can enhance the efficacy of PD-1 therapy and potentially inhibit the progression of adverse reactions, PDT emerges as a critical option for managing severe irAEs following immunotherapy.

We report a case of a 79-year-old female patient with advanced mid-lower esophageal cancer who experienced significant tumor shrinkage following treatment with Tislelizumab. However, after the first cycle of Tislelizumab, the patient developed pneumonia, and after the second cycle, she experienced multi-organ dysfunction, including ptosis, proximal muscle damage, and myocardial injury. Following discontinuation of the PD-1 inhibitor and administration of systemic corticosteroids, the patient’s symptoms improved. Subsequently, PDT was administered for esophageal cancer, leading to significant tumor control and complete resolution of adverse events.

## Case description

**Figure 1:**
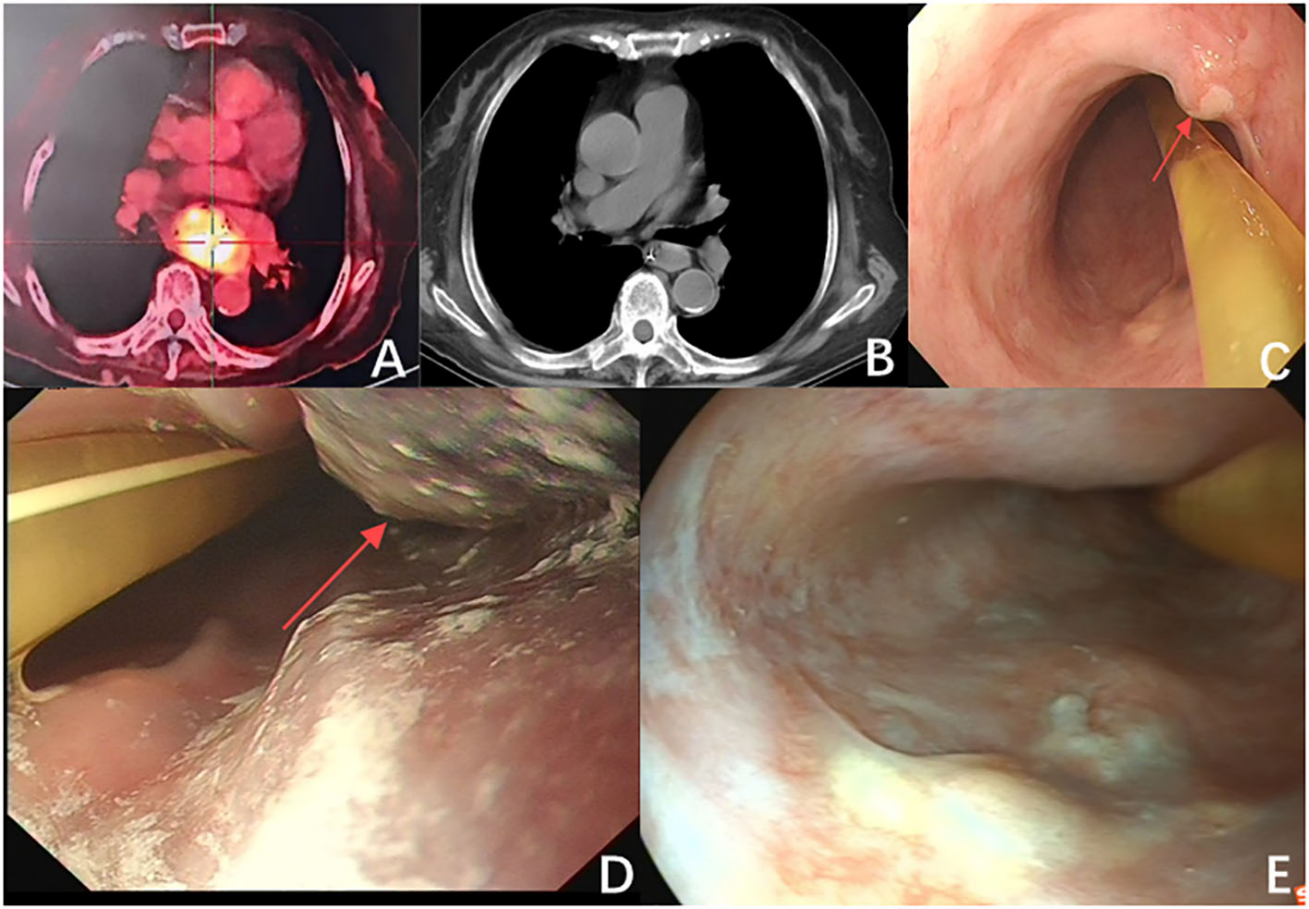
**(A)** The largest cross-section of the tumor before immunotherapy measures 5.5 cm × 3.5 cm (red arrow). **(B)** The tumor size is significantly reduced after immunotherapy (red arrow). **(C)** Endoscopic examination after immunotherapy showed a significant reduction in tumor size, with a diameter of approximately 1.2 cm (red arrow). **(D)** The tumor after the first photodynamic therapy (red arrow). **(E)** The follow-up two months post-treatment indicated that the patient’s tumor showed no significant progression, and there was notable scarring following photodynamic therapy.

**Figure 2:**
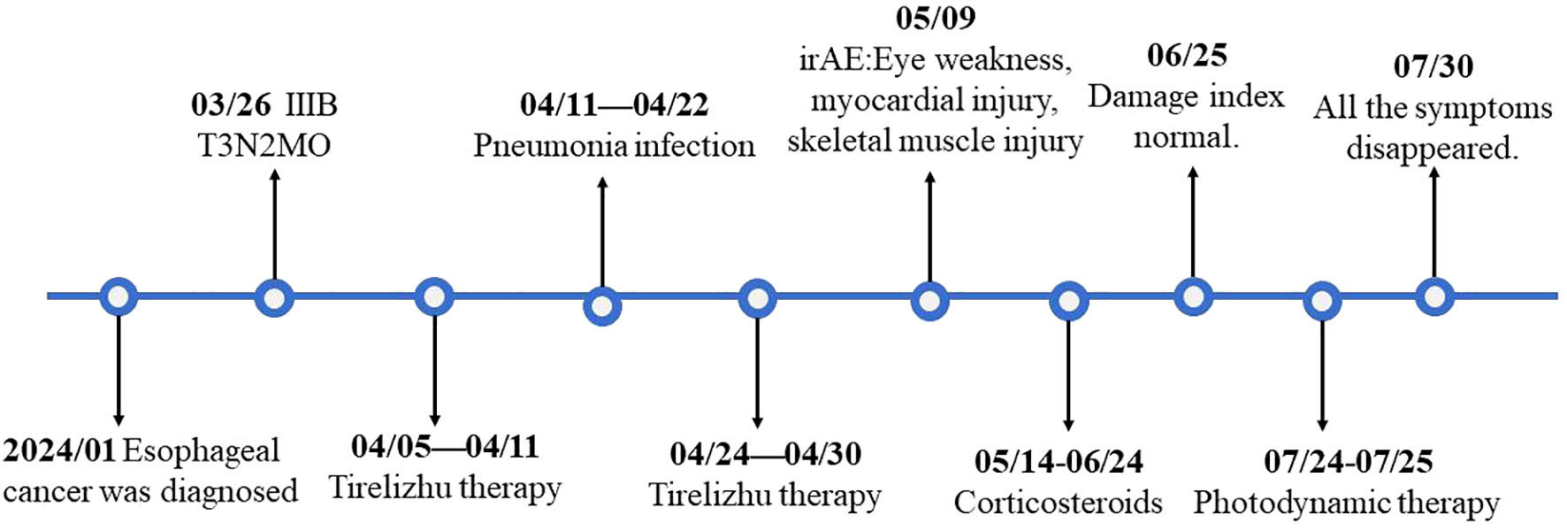
Timetable of case presentation.

A 79-year-old Asian female patient with a history of over 30 years of hypertension, type 2 diabetes, and coronary heart disease presented to our department on March 26, 2024. The patient had been diagnosed with esophageal squamous cell carcinoma following an esophagogastroduodenoscopy and biopsy performed in January 2024 at another hospital. A subsequent computed tomography (CT) scan indicated no metastasis to other sites. Positron emission tomography-computed tomography (PET-CT) showed no metastatic disease(**Figure 1A**), and the surgical staging was determined to be stage IIIB (cT3N2M0). Upon evaluation, the patient exhibited significant dysphagia, dizziness, and fatigue, and had been bedridden for a long time, with a Karnofsky Performance Status (KPS) score of 40. Immunohistochemical analysis revealed CK5/6 and P63 positivity. Due to the absence of surgical indications and the patient’s inability to meet the baseline requirements for chemotherapy, she received monotherapy with the immune checkpoint inhibitor Tislelizumab (200 mg Q21, administered via intravenous infusion). The first cycle of treatment began on April 11, 2024, with injections administered every 21 days. After treatment, the patient developed a significant fever due to a pulmonary infection. The symptoms resolved following intravenous administration of cefoperazone-sulbactam and meropenem. Although the patient was eligible for adjuvant chemotherapy, it was not administered due to the patient’s low KPS score. On April 24, 2024, after the fever subsided, the patient received a second cycle of Tislelizumab (200 mg, intravenous infusion), during which no significant abnormalities were observed (**Figure 2**).

**Figure 3:**
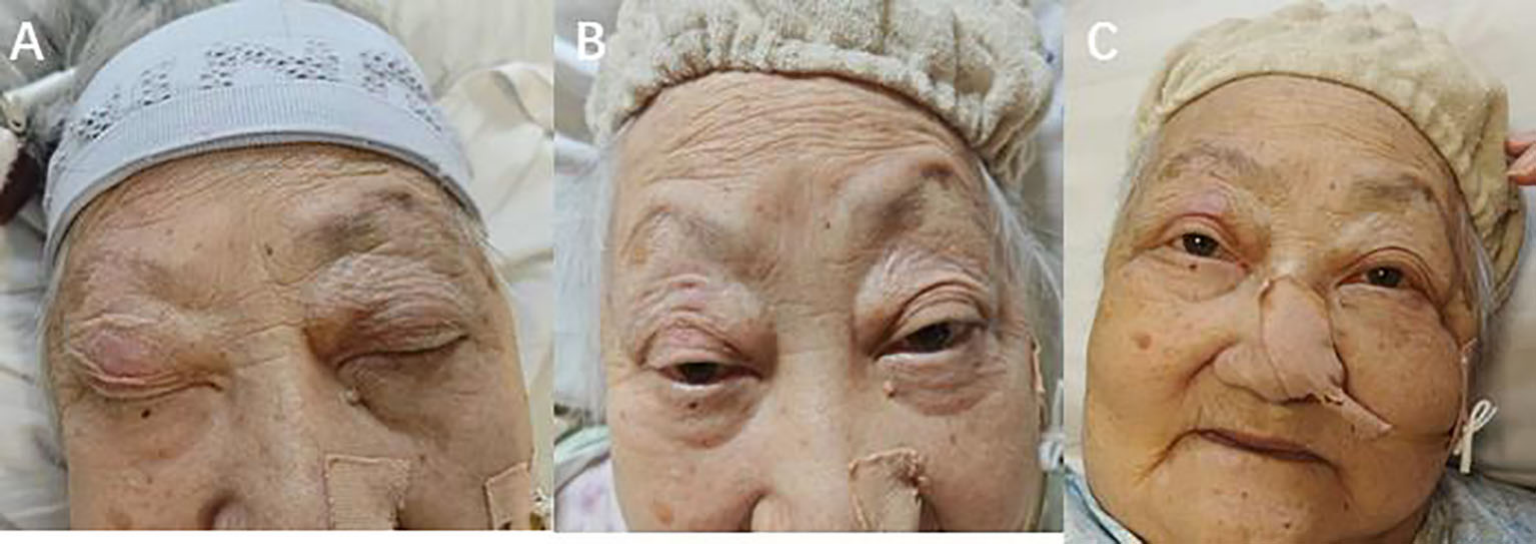
**(A)** The patient suffered from an immune adverse reaction and eyelid weakness. **(B)** The patient’s eyelids returned to normal after photodynamic therapy and corticosteroid therapy **(C)** 2 months after photodynamic therapy, the patient did not experience any adverse reactions.

Unfortunately, after discharge, the patient experienced severe bilateral eyelid ptosis (**Figure 3A**), recurrent palpitations, and upper limb proximal muscle pain. On May 9, 2024, the patient was readmitted to the hospital. Subsequent examinations included an MRI of the brain, as well as assessments of the eyes and heart. The MRI showed no significant changes, the ophthalmological examination indicated no notable vision abnormalities, and the cardiac ultrasound did not reveal any significant worsening of the condition. Since ocular changes and cardiac damage are not common adverse reactions associated with PD-1 inhibitors, differentiation was particularly challenging (4). Further investigations included assessments of the endocrine, circulatory, respiratory systems, and muscle damage. Laboratory results showed elevated MYO: 1428 μg/L (normal range 10-70), BNP: 173 pg/ml (normal range 28.3–37.5), CK: 1127.6 U/L (normal range 40-200), LDH: 604.6 U/L (normal range 109-245), and positive Titin antibody. Electromyography indicated decreased compound muscle action potentials on both sides and peripheral nerve damage in both lower limbs. Given the patient’s severe metabolic disease, which is considered a high-risk factor for irAEs, and taking into account the patient’s medication and medical history, we are inclined to diagnose the irAE as grade 4.

**Figure 4:**
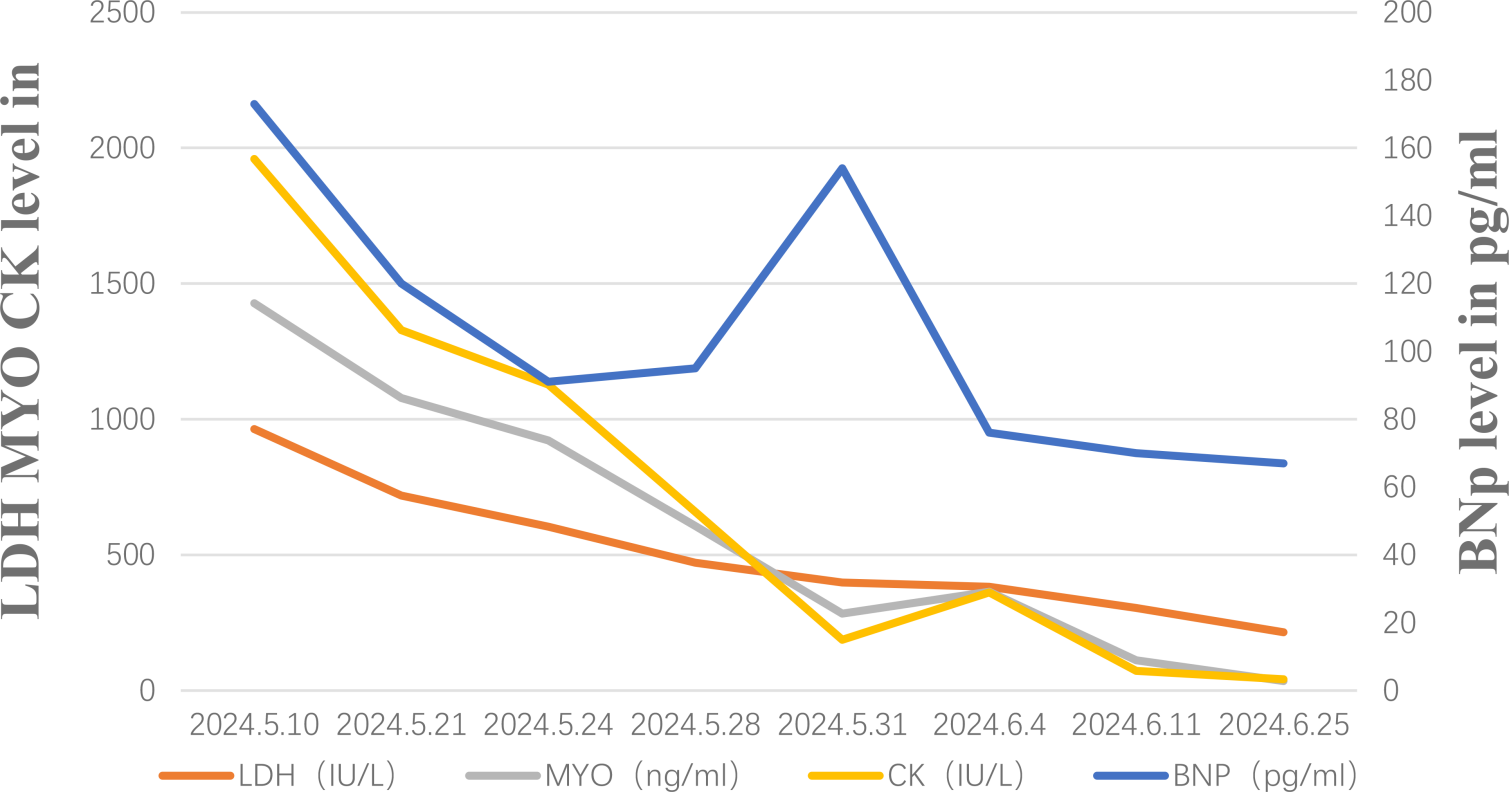
Changes in BNP, LDH, MYO, and CK levels after admission.

The patient started intravenous infusions of 40 mg methylprednisolone sodium succinate for 12 days. After this treatment, the ability to open and close the left eyelid gradually improved, and muscle pain subsided. The regimen was then switched to oral methylprednisolone at a dose of 32 mg, with a taper of 4 mg per week. By June 25, most clinical indicators had returned to normal (**Figure 4**), although there was still some residual muscle weakness in the right eyelid. Following two cycles of Tislelizumab, the tumor showed significant shrinkage compared to initial measurements (**Figure 1B**). However, due to the patient’s intolerance to both chemotherapy and immunotherapy and considering her weakened immune status, localized PDT was selected as the next treatment option (5).

We performed a gastroscopy, which revealed a tumor approximately 1.2 cm in size (**Figure 1C**). After evaluation, we administered Hiporfin at a dose of 2 mg/kg. Forty-eight hours later, on July 24 and 25, photodynamic therapy (Shenzhen Laser Medical Technology, PDT630-A) was performed (**Figure 1D**). Using a 3 cm optical fiber, light irradiation was applied with an energy density of 100 W/cm², delivering 150 J of energy. The duration of irradiation was 900 seconds (630 J) and 1200 seconds (945 J), respectively. Five days later, the patient’s ptosis completely resolved (**Figure 3B**), and all adverse reactions disappeared. We conducted a two-month follow-up with the patient, during which no adverse reactions were observed. After this period, an endoscopic examination revealed no significant tumor progression, and the patient’s condition showed favorable recovery (**Figures 1E**, **3C**).

## Discussion

We describe the case of a patient with advanced esophageal cancer who developed irAEs after receiving two cycles of immune checkpoint inhibitor (ICI) monotherapy with Tislelizumab. While the tumor size was significantly reduced, the patient developed rare bilateral eyelid muscle weakness following the second cycle of ICI treatment, accompanied by cardiac and skeletal muscle injury.

The exact biological mechanisms behind the site and severity of irAE damage with immune checkpoint inhibitors (ICIs) are not fully understood, resulting in significant variability in irAE presentations.PD-1 inhibitors, commonly used among ICIs, tend to have adverse reactions primarily affecting cardiovascular and pulmonary functions, in contrast to CTLA-4 inhibitors, which mainly cause endocrine-related adverse effects (6). Previous literature has documented common irAEs associated with PD-1 inhibitors, such as pneumonia and arthralgia, which aligns with the findings of this study (2). Notably, the patient also experienced myocardial damage, skeletal muscle injury, and eyelid muscle weakness. The incidence of myocardial damage is reported to be <1% (7), and skeletal muscle injury is often associated with myocardial damage, with an incidence of <4% (8). There are few reports documenting adverse reactions such as corneal ulcers associated with PD-1 inhibitors (9), but there have been no reports to date regarding eyelid muscle weakness caused by Tislelizumab.

When PD-1 inhibitors are used, the inhibition of the PD-1/PD-L1 pathway results in the suppression of regulatory T cells (Tregs) and an increase in cytokines (such as TNF, IFN-γ, IL-2, IL-17, CXCL10), which can significantly activate and proliferate T cells, leading to a risk of immune overactivation (2). Compared to other PD-1 inhibitors, Tislelizumab possesses multiple mutations in its Fc hinge region, preventing binding to FcγR and thereby inhibiting the phagocytic activation of macrophages, which reduces T cell clearance (10). This allows for maximal preservation of T cell immunity, but it also results in more severe irAEs. Due to the low tumor-selectivity of T cell immunity activated by PD-1 inhibitors, there is potential for cross-reactivity with normal tissues, resulting in the misidentification of normal tissues as antigens and subsequent immune attacks on them. Prior studies have identified a high frequency of T cell receptor sequences shared between skeletal muscle, myocardium, and tumor infiltrates, suggesting the presence of highly variable complementary determining region 3 (CDR3) sequences (11). Furthermore, the substantial release of cytokines contributes to widespread inflammatory responses, which may also be potential causes of irAEs. However, given the rarity of ocular adverse reactions and the lack of clarity in relevant research, it remains uncertain whether there is a shared receptor influence on ocular irAEs.

Given the unclear mechanisms behind multi-organ irAEs, and the absence of consensus guidelines for managing such cases despite established guidelines for single-organ irAEs (12), treatment largely relies on clinical experience. Early use of corticosteroids is crucial for achieving favorable outcomes in patients. Although immunoglobulin and plasma exchange are also recommended in the guidelines (13), the patients declined these treatments for economic reasons. After administering corticosteroids, their symptoms significantly improved, leading us to discontinue the use of other immunosuppressants, such as cyclophosphamide, for managing irAEs. However, due to the patient’s low KPS score and severe adverse reactions, recovery has been slow, presenting challenges for subsequent esophageal cancer treatment. After re-administration of ICIs, the estimated recurrence rate of adverse reactions is 28.8% (14), making further immune activation therapy deemed unsuitable for this patient. Considering the patient’s physical tolerance and personal wishes, paclitaxel and trastuzumab should only be administered after irAEs have completely resolved and the patient’s condition has improved, making local photodynamic therapy a more feasible option.

Photodynamic therapy may serve as an important adjunctive treatment following ICIs. First, the combination of PD-1 inhibitors and PDT can significantly enhance the immune response against tumors. Second, PDT may alleviate the side effects associated with immunosuppressants. Compared to other adjunctive therapies, photodynamic therapy is more suitable for the current patients. Existing clinical data indicate that photodynamic therapy can significantly inhibit tumor progression and is one of the important palliative treatments for advanced stages of the disease, often serving as adjuvant therapy following radiotherapy, chemotherapy, and immunotherapy (21–24). Recent foundational studies have demonstrated the efficacy of combining ICIs with PDT (18, 20, 25).PDT enhances local dendritic cell cross-presentation of tumor antigens and induces immunogenic cell death (ICD) in tumor cells, thereby mediating CD8+ T cell immune responses (15). This activation is typically suppressed by regulatory T cells (Tregs) (16). The use of ICIs inhibits Treg cells, further enhancing the immune response facilitated by PDT. On the other hand, various photosensitizers used in photodynamic therapy can increase tumor sensitivity to PD-L1 antibody checkpoint inhibition, thereby enhancing the immune response against the tumor (17, 18). The synergistic mechanisms of PDT and ICIs are related to the enrichment of immune cells and the formation of immune memory. Existing studies suggest that their combination may promote immune cell infiltration by altering the expression of ALAS2, ITGA10, and ADAM12 (19). This activation of tumor immune memory can lead to the destruction of tumor cells while simultaneously inhibiting distal tumor growth and preventing metastasis and recurrence (20).

Although PDT does not alter the cross-reactivity of normal tissues due to T cell low immune selectivity, it enhances T cell immune selectivity and directs T cells towards tumor sites, providing potential for controlling irAEs. By inducing immunogenic cell death (ICD) in tumor cells, PDT activates tumor-specific immunity and improves T cell selectivity (26), reducing attacks on normal cells. At the same time, PDT induces acute inflammatory responses, leading to the rapid release of pro-inflammatory mediators and chemokines from cancer cell membranes, damaged endothelial cells, and tumor stroma, including chemokines MIP-2 and KC, as well as pro-inflammatory mediators such as IL-1, IL-6, and TNF. These factors guide neutrophil recruitment to the tumor site and also direct T cells toward inflammatory and tumor regions (27, 28). Some patients may even exhibit a significant decrease in systemic T cell counts following recruitment, leading some studies to suggest that PDT can inhibit excessive systemic immune responses (29). Recent reports indicate that various carriers, including porphyrin-based metal-organic frameworks (pMOFs) and lipid-based (LP) micelle nanocarriers, can effectively reduce irAEs associated with ICIs and reverse resistance to immunotherapy (10, 30, 31). In summary, PDT holds promise as an important adjunctive approach in the treatment of tumors with ICIs.

This paper reports a case of adverse reactions caused by monotherapy with Tislelizumab. The patient received two cycles of Tislelizumab monotherapy. After the second round of ICI treatment, the patient developed rare bilateral eyelid muscle weakness, accompanied by signs of cardiac and skeletal muscle injury. This presents as a rare case of bilateral eyelid muscle weakness. Due to the complexity of malignant tumors, Tislelizumab-induced irAEs are difficult to identify in clinical practice. Early administration of corticosteroids has demonstrated good efficacy, potentially helping to prevent the rapid progression of multi-organ irAEs. Currently, treatment protocols for ICI adverse reactions remain controversial, particularly in conjunction with subsequent tumor therapies. Photodynamic therapy may represent a promising option, as it not only synergistically enhances the efficacy of immunosuppressants in significantly inhibiting tumors but also provides beneficial effects in alleviating ICI-induced adverse reactions. However, this study has a relatively short follow-up period and is limited by its single-case report nature. Future clinical data will be needed to further validate these findings.

## Data Availability

The original contributions presented in the study are included in the article/supplementary material. Further inquiries can be directed to the corresponding author.

## References

[1] Choi YY Kim H Shin S-J Kim HY Lee J Yang H-K . Microsatellite instability and programmed cell death-ligand 1 expression in stage II/III gastric cancer: *post hoc* analysis of the CLASSIC randomized controlled study. Ann Surg. (2019) 270:309–16. doi: 10.1097/SLA.0000000000002803 29727332

[2] Ramos-Casals M Brahmer JR Callahan MK Flores-Chávez A Keegan N Khamashta MA . Immune-related adverse events of checkpoint inhibitors. Nat Rev Dis Primers. (2020) 6:38. doi: 10.1038/s41572-020-0160-6 32382051 PMC9728094

[3] Khoja L Day D Wei-Wu Chen T Siu LL Hansen AR . Tumour- and class-specific patterns of immune-related adverse events of immune checkpoint inhibitors: a systematic review. Ann Oncol. (2017) 28:2377–85. doi: 10.1093/annonc/mdx286 28945858

[4] Chennamadhavuni A Abushahin L Jin N Presley CJ Manne A . Risk factors and biomarkers for immune-related adverse events: A practical guide to identifying high-risk patients and rechallenging immune checkpoint inhibitors. Front Immunol. (2022) 13:779691. doi: 10.3389/fimmu.2022.779691 35558065 PMC9086893

[5] Yano T Wang KK . Photodynamic therapy for gastrointestinal cancer. Photochem Photobiol. (2020) 96:517–23. doi: 10.1111/php.13206 31886891

[6] Grabie N Gotsman I DaCosta R Pang H Stavrakis G Butte MJ . Endothelial programmed death-1 ligand 1 (PD-L1) regulates CD8+ T-cell mediated injury in the heart. Circulation. (2007) 116:2062–71. doi: 10.1161/CIRCULATIONAHA.107.709360 17938288

[7] Patel RP Parikh R Gunturu KS Tariq RZ Dani SS Ganatra S . Cardiotoxicity of immune checkpoint inhibitors. Curr Oncol Rep. (2021) 23:79. doi: 10.1007/s11912-021-01070-6 33937956 PMC8088903

[8] Anquetil C Salem J-E Lebrun-Vignes B Johnson DB Mammen AL Stenzel W . Immune checkpoint inhibitor-associated myositis: expanding the spectrum of cardiac complications of the immunotherapy revolution. Circulation. (2018) 138:743–5. doi: 10.1161/CIRCULATIONAHA.118.035898 30359135

[9] Xiang X Lin W Guan X Zhou B Yuan Y Silva D . Corneal ulcer development due to sintilimab-anlotinib combination therapy-induced dry eye: a case report. Transl Cancer Res. (2024) 13:2571–9. doi: 10.21037/tcr-23-1952 PMC11170519 38881937

[10] Zhang L Geng Z Hao B Geng Q . Tislelizumab: A modified anti-tumor programmed death receptor 1 antibody. Cancer Control. (2022) 29:10732748221111296. doi: 10.1177/10732748221111296 35926155 PMC9358212

[11] Cheng F Loscalzo J . Autoimmune cardiotoxicity of cancer immunotherapy. Trends Immunol. (2017) 38:77–8. doi: 10.1016/j.it.2016.11.007 27919707

[12] Puzanov I Diab A Abdallah K Bingham CO Brogdon C Dadu R . Managing toxicities associated with immune checkpoint inhibitors: consensus recommendations from the Society for Immunotherapy of Cancer (SITC) Toxicity Management Working Group. J Immunother Cancer. (2017) 5:95. doi: 10.1186/s40425-017-0300-z 29162153 PMC5697162

[13] Brahmer JR Abu-Sbeih H Ascierto PA Brufsky J Cappelli LC Cortazar FB . Society for Immunotherapy of Cancer (SITC) clinical practice guideline on immune checkpoint inhibitor-related adverse events. J Immunother Cancer. (2021) 9:e002435. doi: 10.1136/jitc-2021-002435 34172516 PMC8237720

[14] Dolladille C Ederhy S Sassier M Cautela J Thuny F Cohen AA . Immune checkpoint inhibitor rechallenge after immune-related adverse events in patients with cancer. JAMA Oncol. (2020) 6:865–71. doi: 10.1001/jamaoncol.2020.0726 PMC7163782 32297899

[15] Canti G Calastretti A Bevilacqua A Reddi E Palumbo G Nicolin A . Combination of photodynamic therapy + immunotherapy + chemotherapy in murine leukiemia. Neoplasma. (2010) 57:184–8. doi: 10.4149/neo_2010_02_184 20099984

[16] Kleinovink JW Fransen MF Löwik CW Ossendorp F . Photodynamic-immune checkpoint therapy eradicates local and distant tumors by CD8+ T cells. Cancer Immunol Res. (2017) 5:832–8. doi: 10.1158/2326-6066.CIR-17-0055 28851692

[17] Duan X Chan C Guo N Han W Weichselbaum RR Lin W . Photodynamic therapy mediated by nontoxic core-shell nanoparticles synergizes with immune checkpoint blockade to elicit antitumor immunity and antimetastatic effect on breast cancer. J Am Chem Soc. (2016) 138:16686–95. doi: 10.1021/jacs.6b09538 PMC5667903 27976881

[18] Zhou Y Zhang W Wang B Wang P Li D Cao T . Mitochondria-targeted photodynamic therapy triggers GSDME-mediated pyroptosis and sensitizes anti-PD-1 therapy in colorectal cancer. J Immunother Cancer. (2024) 12:e008054. doi: 10.1136/jitc-2023-008054 38429070 PMC10910688

[19] Gong B Wang L Zhang H Wang Q Li W . Amplifying T cell-mediated antitumor immune responses in nonsmall cell lung cancer through photodynamic therapy and anti-PD1. Cell Biochem Funct. (2024) 42:e3925. doi: 10.1002/cbf.3925 38269509

[20] Lou J Aragaki M Bernards N Chee T Gregor A Hiraishi Y . Repeated photodynamic therapy mediates the abscopal effect through multiple innate and adaptive immune responses with and without immune checkpoint therapy. Biomaterials. (2023) 292:121918. doi: 10.1016/j.biomaterials.2022.121918 36442438

[21] Luketich JD Christie NA Buenaventura PO Weigel TL Keenan RJ Nguyen NT . Endoscopic photodynamic therapy for obstructing esophageal cancer: 77 cases over a 2-year period. Surg Endosc. (2000) 14:653–7. doi: 10.1007/s004640000144 10948303

[22] Wang X-Y Maswikiti EP Zhu J-Y Ma Y-L Zheng P Yu Y . Photodynamic therapy combined with immunotherapy for an advanced esophageal cancer with an obstruction post metal stent implantation: A case report and literature review. Photodiagnosis Photodyn Ther. (2022) 37:102671. doi: 10.1016/j.pdpdt.2021.102671 34864195

[23] Rupinski M Zagorowicz E Regula J Fijuth J Kraszewska E Polkowski M . Randomized comparison of three palliative regimens including brachytherapy, photodynamic therapy, and APC in patients with Malignant dysphagia (CONSORT 1a) (Revised II). Am J Gastroenterol. (2011) 106:1612–20. doi: 10.1038/ajg.2011.178 21670770

[24] Yano T Muto M Minashi K Iwasaki J Kojima T Fuse N . Photodynamic therapy as salvage treatment for local failure after chemoradiotherapy in patients with esophageal squamous cell carcinoma: a phase II study. Int J Cancer. (2012) 131:1228–34. doi: 10.1002/ijc.27320 22024814

[25] Hao Y Chung CK Gu Z Schomann T Dong X Veld RVHI‘t . Combinatorial therapeutic approaches of photodynamic therapy and immune checkpoint blockade for colon cancer treatment. Mol BioMed. (2022) 3:26. doi: 10.1186/s43556-022-00086-z 35974207 PMC9381671

[26] Brackett CM Gollnick SO . Photodynamic therapy enhancement of anti-tumor immunity. Photochem Photobiol Sci. (2011) 10:649–52. doi: 10.1039/c0pp00354a PMC3197776 21253659

[27] New JY Li B Koh WP Ng HK Tan SY Yap EH . T cell infiltration and chemokine expression: relevance to the disease localization in murine graft-versus-host disease. Bone Marrow Transplant. (2002) 29:979–86. doi: 10.1038/sj.bmt.1703563 12098066

[28] Jalili A Makowski M Switaj T Nowis D Wilczynski GM Wilczek E . Effective photoimmunotherapy of murine colon carcinoma induced by the combination of photodynamic therapy and dendritic cells. Clin Cancer Res. (2004) 10:4498–508. doi: 10.1158/1078-0432.CCR-04-0367 15240542

[29] Jolles CJ Ott MJ Straight RC Lynch DH . Systemic immunosuppression induced by peritoneal photodynamic therapy. Am J Obstet Gynecol. (1988) 158:1446–53. doi: 10.1016/0002-9378(88)90380-8 2968047

[30] Chen Q He Y Wang Y Li C Zhang Y Guo Q . Penetrable nanoplatform for “Cold” Tumor immune microenvironment reeducation. Adv Sci (Weinh). (2020) 7:2000411. doi: 10.1002/advs.202000411 32995118 PMC7503208

[31] Huang J Xiao Z An Y Han S Wu W Wang Y . Nanodrug with dual-sensitivity to tumor microenvironment for immuno-sonodynamic anti-cancer therapy. Biomaterials. (2021) 269:120636. doi: 10.1016/j.biomaterials.2020.120636 33453632

